# Impact of Net Atrioventricular Compliance on Mitral Valve Area Assessment—A Perspective Considering Three-Dimensional Mitral Valve Area by Transesophageal Echocardiography

**DOI:** 10.3390/diagnostics14151595

**Published:** 2024-07-24

**Authors:** Tony Li, Ryan Leow, Meei Wah Chan, William K. F. Kong, Ivandito Kuntjoro, Kian Keong Poh, Ching Hui Sia, Tiong Cheng Yeo

**Affiliations:** 1 Department of Cardiology, National University Heart Centre Singapore, 1E Kent Ridge Road, Tower Block Level 9, Singapore 119228, Singaporeching_hui_sia@nuhs.edu.sg (C.H.S.); 2 Department of Medicine, Yong Loo Lin School of Medicine, National University of Singapore, Singapore 117597, Singapore

**Keywords:** mitral stenosis, rheumatic heart disease, pressure half-time, net atrioventricular compliance

## Abstract

Background: Net atrioventricular compliance (C_n_) can affect the accuracy of mitral valve area (MVA) assessment. We assessed how different methods of MVA assessment are affected by C_n_, and if patients with abnormal C_n_ may be identified by clinical and/or echocardiographic parameters. Methods: We studied 244 patients with rheumatic MS. The concordance between mitral valve area (MVA) by 2D planimetry, pressure half-time (PHT), continuity equation (CE), Yeo’s index, and 3-dimensional mitral valve area assessed by transesophageal echocardiography (TEE 3DMVA) in patients with normal and abnormal C_n_ (C_n_ ≤ 4 mL/mmHg) were evaluated in the 110 patients with both transesophageal echocardiogram (TEE) and transthoracic echocardiogram (TTE). Variables that were associated with abnormal C_n_ were validated in the remaining 134 patients with only TTE. Results: Except for MVA by CE, concordance with TEE 3DMVA was poorer for all other methods of MVA assessment in patients with abnormal C_n_. But, the difference in concordance was only statistically significant for MVA by PHT. Patients with MVA ≤ 1.5 cm^2^ by 2D planimetry and PHT ≤ 130 ms were likely to have an abnormal C_n_. (specificity 98.5%). This finding was validated in the remaining 134 patients (specificity 93%). Conclusions: MVA assessment by PHT is significantly affected by C_n_. Abnormal C_n_ should be suspected when 2D planimetry MVA is ≤1.5 cm^2^ together with an inappropriately short PHT that is ≤130 ms. In this scenario, MVA by PHT is inaccurate.

## 1. Introduction

Mitral stenosis (MS) is the most common manifestation of rheumatic heart disease with gradual stenosis of the valve leading to deleterious consequences over time [1,2]. The assessment of MS severity is primarily based on measurement of the mitral valve area (MVA) on transthoracic echocardiography (TTE) [3]. This can be accomplished using direct anatomic or indirect hemodynamic assessment. The technique of 2D planimetry allows for direct anatomical measurement of the MVA and has been shown to have good agreement with findings from surgically excised mitral valves [4,5]. Hemodynamic assessment of MVA includes the pressure half-time (PHT), continuity equation (CE), and proximal isovelocity surface area (PISA) methods [6,7,8,9,10]. We recently reported that Yeo’s index was able to reliably identify MS of varying severity using direct anatomical assessment from the mitral leaflet separation index (MLSI) together with hemodynamic assessment with the dimensionless index (DI) and verified its accuracy in comparison to 3-dimensional MVA on transesophageal echocardiography (TEE 3DMVA) and in mixed valvular disease [11,12,13]. 

Net atrioventricular compliance (C_n_) is a parameter that can express the compliance of both the atrium and ventricle. While it was initially derived invasively, studies have shown that C_n_ can be derived non-invasively from echocardiographic transmitral mitral profiles using the formula $C_{n}=\frac{1270\times MVA\;by\;2D\;planimetry}{E-wave\;downslope}$, with good correlation to invasively determined values [14,15]. C_n_ is a predictor of valvular intervention and cardiovascular mortality in patients with MS [16,17]. Nunes et al. showed that C_n_ ≤ 4 mL/mmHg predicted unfavorable outcomes of either MV intervention or death [16]. Studies also found that abnormal C_n_ could lead to under-estimation of the MVA by PHT as compared to MVA by 2D planimetry [18,19].

The aim of this study is to assess how different methods of MVA assessment were affected by C_n_, and if patients with abnormal C_n_ could be identified by clinical or echocardiographic parameters.

## 2. Methodology

### 2.1. Study Population

This study evaluated 244 patients (110 patients who had both TTE and TEE with 3D MVA measurement, and 134 patients who only had TTE) at a tertiary center. The study was approved by the National Healthcare Group Institutional Review Board (NHG DSRB 2021/00603). Echocardiographic data and relevant clinical information were obtained from the electronic medical records and databases.

### 2.2. Echocardiographic Assessment

TEE 3DMVA was assessed using the 3D echocardiographic imaging platform (iE33; Philips Medical Systems, Andover, MA, USA) and a 3D TEE probe (X-9; Philips Medical Systems). TEE 3DMVA was measured using multiplanar reconstruction or direct planimetry of the narrowest mitral valve orifice. The TTE study performed within 1 year of the TEE study was identified from the echocardiography database for analysis of Yeo’s index and measurement of MVA using 2D planimetry, PHT, and CE methods. Yeo’s index was determined as the product of the mitral leaflet separation index (MLSI) and the dimensionless index (DI) [11]. The maximal diastolic separation of the mitral valve leaflet tips was assessed in the parasternal long-axis view and the apical four-chamber view and the average was taken to be the MLSI [20]. A mean of 3 measurements was taken for patients in sinus rhythm while 5 measurements were taken in patients with atrial fibrillation. The DI was calculated by dividing the left ventricular outflow tract (LVOT) PW Doppler TVI by the MV CW Doppler TVI. MVA measurements using 2D planimetry, PHT, and CE were performed in accordance to consensus guidelines [3,10]. 

### 2.3. Assessment of C_n_

C_n_ was determined non-invasively by means of Doppler echocardiography in accordance to previously described methods using the formula $C_{n}=\frac{1270\times MVA\;by\;2D\;planimetry}{E-wave\;downslope}$ [14,15]. In patients with non-linear diastolic flow, the mid-diastolic flow was used to measure the E-wave downslope.

### 2.4. Statistical Analysis

C_n_ ≤ 4 mL/mmHg was considered as abnormal net atrioventricular compliance [16]. In the 110 patients who had both TTE and TEE, Yeo’s index and MVA derived by 2D planimetry, PHT, and CE were assessed for concordance with TEE 3DMVA in patients with normal and abnormal C_n_ using the intra-class correlation coefficients (*ρ*_c_) [21]. 

For concordance analysis, cases were divided into non-severe (MVA > 1.5 cm^2^), severe (1.0 cm^2^ < MVA ≤ 1.5 cm^2^), and very severe (MVA ≤ 1.0 cm^2^) MS. Yeo’s index ≤ 0.26 cm and ≤0.15 cm were considered as synonymous with MVA ≤ 1.5 cm^2^ and ≤1.0 cm^2^, respectively [11]. Difference in concordance between patients with normal C_n_ and abnormal C_n_ was considered significant if there was no overlap in the 95% confidence intervals of the intra-class correlation coefficients. Univariable analysis of clinical and echocardiographic parameters was performed to identify predictors of abnormal C_n_. The identified predictors were then validated in the remaining 134 patients who had only TTE. 

Continuous variables were expressed as mean (±standard deviation), while categorical variables were expressed as a number (proportion). Statistical analysis was performed with IBM SPSS Statistics Version 26 (IBM Corp., Armonk, NY, USA) and MedCalc Statistical Software version 19.2.6 (MedCalc Software bv, Ostend, Belgium; https://www.medcalc.org; 2020). *p*-values were 2-sided and deemed significant if <0.05.

## 3. Results

### 3.1. Cohort Characteristics

The clinical and echocardiographic characteristics, comorbidities, and medications of the cohort are shown in Table 1. For the TEE cohort, the mean age was 62.3 (±12.7) years and 81 patients (73.0%) were female. The most common race was Chinese followed by Indian and Malay. There was a small proportion of other South Asian ethnicities with only one Caucasian patient. A substantial 60 patients (54.1%) had atrial fibrillation at the time of the index echocardiogram, and 44 patients (39.6%) had hypertension, while 16 patients (14.4%) had ischemic heart disease, out of which nine had a prior myocardial infarction and percutaneous coronary intervention. There were 15 patients with prior stroke, out of whom all but three had atrial fibrillation. In terms of medications, 49.5% of the patients were on oral anticoagulation while 22.5% were on some form of antiplatelets, predominantly aspirin; there were three patients on dual antiplatelet therapy. There were 28 (25.2%) patients on diuretics, which was most commonly a loop diuretic. The use of ACE inhibitors or ARBs was relatively common (13.5%) but there was no patient on ARNI. Only two patients were on SGLT2 inhibitors. MVA by CE was the smallest while MVA by PHT was the largest. Forty-six patients (41.8%) had abnormal C_n_. The median time between TTE and TEE was 28 (interquartile range 7–162) days. The validation cohort had a mean age of 54.1 (±12.0) years and 93 patients (63.4%) were female. 

### 3.2. Concordance Analysis in Relation to C_n_

The results of concordance analysis between TEE 3DMVA and Yeo’s index, MVA by 2D planimetry, CE, and PHT methods using TEE 3DMVA as gold standard are shown in Table 2. Overall, concordance was the best with Yeo’s index followed by 2D planimetry, CE, and PHT. In patients with abnormal C_n_, concordance was the best with CE, followed by Yeo’s index, 2D planimetry, and PHT. Except for MVA by CE, concordance between TEE 3DMVA and all the other methods of MVA assessment was poorer in patients with abnormal C_n_ compared with patients with normal C_n_. Interestingly, an inverse relation was observed for MVA by CE where performance was better (*ρ*_c_ = 0.659 vs. *ρ*_c_ = 0.464) in cases of abnormal C_n_. However, the difference in concordance between patients with abnormal and normal C_n_ was only statistically significant for MVA by PHT. 

### 3.3. Predictors of Abnormal C_n_

As shown in Table 3, there was no significant difference in clinical parameters including age, sex, BMI, or other comorbidities in patients with abnormal and normal C_n_. Amongst echocardiographic parameters, it was found that MVA by 2D planimetry ≤ 1.5 cm^2^ and PHT ≤ 130 ms were associated with an abnormal C_n_. A situation where both 2D MVA ≤ 1.5 cm^2^ and PHT ≤ 130 ms were present was highly specific for an abnormal C_n_ (98.5%). When tested in the 134 patients with only TTE, the presence of MVA ≤ 1.5 cm^2^ together with PHT ≤ 130 ms had a specificity of 93% for identifying patients with abnormal C_n_. This is shown in Table 4. 

## 4. Discussion

C_n_ has been studied as a parameter that can reflect changes in atrial and ventricular compliance. While other studies have pointed out that changes in C_n_ can affect the transmitral flow and hence may impact the accuracy of MVA assessment on TTE, they used 2D planimetry as the reference standard [18,19]. To our knowledge, this is the first study to evaluate the impact of C_n_ on the accuracy of MVA assessment using TEE 3DMVA as the gold standard measure for MS severity.

### 4.1. Different Modes of MVA Assessment

Despite advancements in transthoracic echocardiography, there remains no gold standard on TTE for the assessment of MS severity as each of the currently available methods has its own limitations. The method of 2D planimetry relies on direct visualization of the flow-limiting MV orifice in the parasternal short-axis view which is operator dependent and can be technically challenging. The CE method requires multiple measurements which can each introduce error [22]. Furthermore, it is inaccurate in the presence of significant mitral or aortic regurgitation [23]. The PHT method has been shown to be inaccurate in numerous situations including tachycardia, atrial fibrillation, aortic regurgitation, and non-linear Doppler velocity tracings [18,19,24]. The current gold standard for assessment of MVA has pivoted to 3D MVA assessment on TEE [25,26]. 

This study shows the following:

1. MVA assessment by PHT is significantly affected by net atrioventricular compliance;
2. Except for MVA by CE, concordance with TEE 3DMVA was poorer for all other methods of MVA assessment in patients with abnormal C_n_ compared to those with normal C_n_;
3. Abnormal C_n_ should be suspected when 2D planimetry MVA is ≤1.5 cm^2^ together with an inappropriately short PHT that is ≤130 ms. In these patients, MVA by the PHT method is inaccurate and should not be used.

### 4.2. Concordance in Patients with Normal and Abnormal C_n_

Except for CE, the concordance between different methods of MVA assessment and TEE 3DMVA was higher in patients with normal C_n_ compared with patients with abnormal C_n_. However, the difference was only statistically significant for MVA by PHT. Our results suggests that PHT is inaccurate in patients with abnormal C_n_. The PHT method was first proposed by Hatle et al. where the MVA can be estimated from the equation ${MVA}_{PHT}=\frac{220}{PHT}\;$ [6]. This was based on the hypothesis that a narrower mitral valve orifice will restrict transmitral flow and thereafter lead to a more prolonged transmitral flow and thus longer pressure half-time. This was an elegant and simple expression of the hemodynamic significance of MS and has made itself into contemporary echocardiographic guidelines. However, on top of physical obstruction from a stenosed mitral valve orifice, left-sided chamber pressures and compliance can also influence the transmitral flow. For a given MVA, a higher left atrial pressure and lower left ventricular compliance would lead to more rapid mitral deceleration and a shorter pressure half-time and consequently overestimation of the MVA. 

An interesting finding of our study is that concordance between MVA by CE and TEE 3DMVA was higher in patients with abnormal C_n_ compared to patients with normal C_n_. The reason for this is unclear. A previous study using non-invasively determined C_n_ showed that MVA by CE was not significantly affected by changes in C_n_ and may be preferable to the pressure half-time method [27]. However, the sample size was small with only 17 patients.

### 4.3. Predictors of Abnormal C_n_

It might be useful if we can identify patients with an abnormal C_n_ so that methods of MVA assessment with better concordance such as CE, Yeo’s index, or planimetry can be used to assess severity of MS in these patients. However, the calculation of C_n_ can be tedious in everyday practice and having easier means to identify such cases can be helpful. Intuitively, ageing or comorbidities such as ischemic heart disease or atrial fibrillation might lead to atrial or ventricular remodeling and thus an abnormal C_n_. There have also been studies pointing to the influence of extrinsic mechanical factors, particularly anterior chest wall deformities such as concave-shaped chest wall and/or various degrees of pectus excavatum that are commonly associated with tachycardia, impaired diastolic filling, and abnormal biventricular and atrioventricular compliance [28]. However, clinical parameters including age, sex, or comorbidities did not demonstrate a significant association with an abnormal C_n_. Instead, we found that 2D planimetry MVA ≤ 1.5 cm^2^ together with an inappropriately short PHT ≤ 130 ms have a high specificity for abnormal C_n_. This was validated in a separate cohort of patients with MS showing a high specificity of 93% for the presence of abnormal C_n_. 

### 4.4. Clinical Implications

Each of the current echocardiographic methods of MVA assessment has its own limitations. Hence, it is recommended that clinicians use a multi-parametric approach incorporating these echocardiographic methods and considering the patient’s clinical status when managing patients with MS. However, it is not uncommon that these different echocardiographic methods of MVA assessment yield conflicting results. In this instance, if abnormal Cn is present, our findings suggest that MVA by PHT is inaccurate. Instead, MVA assessment by CE, Yeo’s index, or planimetry might be more accurate. In the study by Omar et al., the PISA method showed good correlation with 2D planimetry MVA in cases with extreme C_n_ values [18]. However, the PISA method is not commonly used due to the need for a mitral leaflet angle correction factor, ⍺, which needs to be manually measured, limiting the practicality of this method [29]. Furthermore, the reference standard used in that study was 2D planimetry which is not the gold standard measure of MVA, unlike TEE 3DMVA that is used in this study. It may not be practical to calculate C_n_ in all patients with MS. This study showed that abnormal C_n_ should be suspected when 2D planimetry MVA is ≤1.5 cm^2^ together with an inappropriately short PHT that is ≤130 msec. While the specificity of this combination for identifying low C_n_ is high, its sensitivity is low. Hence, there is still no simple, user-friendly, and reliable method to identify patients with abnormal C_n_.

### 4.5. Limitations

There are several limitations of our study. The findings of this study do not apply to degenerative MS which is growing in prevalence especially with an ageing population. C_n_ measurements were not performed by invasive cardiac assessments. However, invasive cardiac assessment for MS is rarely performed nowadays and studies have shown that echocardiographic assessment of C_n_ correlates well with invasive measurement of C_n_. 

## 5. Conclusions

MVA assessment by PHT was significantly affected by net atrioventricular compliance. Abnormal C_n_ should be suspected when 2D planimetry MVA is ≤1.5 cm^2^ together with an inappropriately short PHT ≤ 130 ms. In this scenario, MVA by PHT is inaccurate and should not be used. Alternative echocardiographic measures with better accuracy should be considered such as Yeo’s index, 2D planimetry, or continuity equation. Yeo’s index is a useful adjunct to existing measures of MS severity and can be considered a promising addition to the repertoire of routine echocardiographic parameters for assessment of MS severity [11,12,13].

## Figures and Tables

**Table 1:** Clinical and echocardiographic characteristics.

	Overall Cohort (*n* = 244)	TEE Cohort (*n* = 110)	Validation Cohort (*n* = 134)
Baseline Characteristics			
Age at diagnosis (years)	57.9 (±13.0) years	62.3 (±12.7) years	54.1 (±12.0) years
Sex (female) (*n*, %)	174 (71.3%)	81 (73.0%)	93 (69.4%)
Ethnicity (*n*, %)			
Chinese	150 (61.5%)	70 (63.6%)	80 (59.7%)
Malay	41 (16.8%)	15 (13.6%)	26 (19.4%)
Indian	26 (10.7%)	16 (14.6%)	10 (7.5%)
Others	27 (11.0%)	9 (8.2%)	18 (13.4%)
Body mass index (kg/m^2^)	24.7 ± 5.60	25.02 ± 5.99	24.34 ± 5.29
Blood pressure (mmHg)	129.0 (±20.8)/71.1 (±11.3)	128.6 (±21.5)/70.4 (±11.2)	127.2 (±20.3)/71.6 (±11.4)
Past Medical History			
Hypertension (%)	101 (41.4%)	44 (39.6%)	57 (42.5%)
Hyperlipidemia (%)	82 (33.6%)	37 (33.3%)	45 (33.6%)
Diabetes mellitus (%)	50 (20.5%)	18 (16.2%)	32 (23.9%)
Ischemic heart disease (%)	37 (15.2%)	16 (14.4%)	21 (15.7%)
Prior myocardial infarction (%)	16 (6.6%))	9 (8.2%)	7 (5.2%)
Chronic kidney disease (%)	19 (7.8%)	6 (5.4%)	13 (9.7%)
Peripheral vascular disease (%)	6 (2.5%)	0 (0.0%)	6 (4.5%)
Atrial fibrillation (%)	129 (52.9%)	60 (54.1%)	69 (51.5%)
Stroke (%)	31 (12.7%)	15 (13.5%)	16 (11.9%)
Medication Use			
Antiplatelet	75 (30.7%)	25 (22.5%)	42 (31.3%)
Oral anticoagulation	114 (46.7%)	55 (49.5%)	59 (44.0%)
Beta blocker	123 (50.4%)	61 (55.0%)	62 (46.3%)
ACE inhibitor/ARB	54 (22.1%)	15 (13.5%)	39 (29.1%)
Calcium channel blocker	25 (10.2%)	11 (9.9%)	14 (10.4%)
Diuretics	60 (24.6%)	28 (25.2%)	32 (23.9%)
Echocardiographic Findings			
LVEF	58.2 ± 8.70	57.13 ± 6.09	58.54 ± 9.4
Mean transmitral gradient	7.24 ± 3.76	6.09 ± 3.98	6.68 ± 3.59
MVA by 2D planimetry	1.31 ± 0.41	1.18 ± 0.38	1.41 ± 0.42
MVA by PHT	1.46 ± 0.53	1.31 ± 0.44	1.58 ± 0.56
MVA by CE	1.06 ± 0.46	0.91 ± 0.32	1.16 ± 0.51
Yeo’s index	0.26 ± 0.16	0.19 ± 0.11	0.33 ± 0.18
C_n_	4.97 ± 1.86	4.43 ± 1.98	5.53 ± 2.74
Abnormal C_n_ ≤ 4	86 (35.2%)	46 (41.8%)	40 (29.9%)

Note: 2D planimetry, two-dimensional planimetry; ACE inhibitor, angiotensin-converting enzyme inhibitor; ARB, aldosterone receptor blocker; CE, continuity equation; C_n_, net atrioventricular compliance; LVEF, left ventricular ejection fraction; MVA, mitral valve area; PHT, pressure half-time.

**Table 2:** Concordance of TTE and TEE assessments stratified by C_n_.

		2D Planimetry	MVA by CE	MVA by PHT	Yeo’s Index
Overall Concordance	Overall	*ρ*_c_ = 0.675 (0.560–0.74)	*ρ*_c_ = 0.464 (0.278–0.592)	*ρ*_c_ = 0.366 (0.235–0.523)	*ρ*_c_ = 0.739 (0.686–0.839)
	Abnormal C_n_ ≤ 4	*ρ*_c_ = 0.593 (0.355–0.747)	*ρ*_c_ = 0.659 (0.420–0.795)	*ρ*_c_ = 0.284 (0.101–0.387)	*ρ*_c_ = 0.646 (0.440–0.788)
	Normal C_n_ > 4	*ρ*_c_ = 0.679 (0.486–0.769)	*ρ*_c_ = 0.323 (0.094–0.519)	*ρ*_c_ = 0.581 (0.396–0.721)	*ρ*_c_ = 0.800 (0.693–0.873)

Note: 2D planimetry, two-dimensional planimetry; CE, continuity equation; C_n_, net atrioventricular compliance; MVA, mitral valve area; PHT, pressure half-time.

**Table 3:** Univariate analysis of factors predicting abnormal C_n_.

	Normal C_n_ (*n* = 65)	Abnormal C_n_ (*n* = 45)	*p*-Value
Clinical Characteristics			
Age	61.49 ± 12.69	63.32 ± 12.71	0.459
Sex	49 (75.3%)	32 (71.1%)	0.427
Body mass index	25.81 ± 6.21	24.10 ± 5.44	0.140
Comorbidities			
Hypertension	27 (41.6%)	17 (37.7%)	0.391
Hyperlipidemia	25 (38.5%)	12 (26.7%)	0.223
Diabetes mellitus	9 (13.8%)	9 (20.0%)	0.438
Ischemic heart disease	9 (13.8%)	7 (15.6%)	0.791
Atrial fibrillation	35 (53.8%)	25 (55.6%)	0.859
History of heart failure	13 (20.0%)	12 (26.7%)	0.490
Echocardiographic Features			
LVEF	58.52 ± 6.23	55.12 ± 7.08	0.229
PHT	186.49 ± 0.55	179.75 ± 62.77	0.552
PHT < 130 ms	4 (6.2%)	9 (20.0%)	0.036
2D MVA	1.32 ± 0.36	0.98 ± 0.30	<0.001
2D MVA ≤ 1.5 cm^2^	54 (83.1%)	44 (97.8%)	0.026

Note: 2D planimetry, two-dimensional planimetry; C_n_, net atrioventricular compliance; LVEF, left ventricular ejection fraction; MVA, mitral valve area; PHT, pressure half-time.

**Table 4:** Assessment of MVA ≤ 1.5 cm^2^ and/or PHT ≤ 130 s for identification of abnormal C_n_.

	*p*-Value	Sensitivity	Specificity
In TEE Cohort			
2D MVA ≤ 1.5 cm^2^ and PHT ≤ 130 ms	*p* = 0.003	17.78%	98.46%
In Validation Cohort			
2D MVA ≤ 1.5 cm^2^ and PHT ≤ 130 ms	*p* < 0.001	30.00%	92.55%

C_n_, net atrioventricular compliance; MVA, mitral valve area; PHT, pressure half-time.

## Data Availability

The data presented in this study are available on request from the corresponding author.
